# Intergenerational transmission of psychopathology across three generations: the role of social support

**DOI:** 10.1007/s00787-024-02562-z

**Published:** 2024-08-17

**Authors:** Yllza Xerxa, Manon H. J. Hillegers, Esther Mesman, Henning Tiemeier, Pauline W. Jansen

**Affiliations:** 1 Department of Child and Adolescent Psychiatry/Psychology, Erasmus University Medical Center, PO Box 2040, 3000 CA Rotterdam, the Netherlands; 2 The Generation R Study Group, Erasmus University Medical Center, Rotterdam, the Netherlands; 3 Department of Social and Behavioral Sciences, Harvard TH Chan School of Public Health, Boston, USA; 4 Department of Psychology, Education and Child Studies, Erasmus University Rotterdam, Rotterdam, the Netherlands

**Keywords:** Social factors, Additive interaction effect, Psychopathology, Intergenerational transmission

## Abstract

Psychopathology runs in families and affects functioning of individuals and their family members. This study assessed the intergenerational transmission of psychopathology risk across three generations, and the extent to which social support factors may protect against this transmission from parents to their offspring. This study was embedded in Generation R, a multi-ethnic population-based cohort from fetal life onwards. Lifetime psychiatric disorders of grandparents were assessed with the Family Informant Schedule Criteria- updated for DSM-IV. Parental psychopathology was repeatedly measured by the Brief Symptom Inventory. Offspring psychopathology (ages 10 and 14) was assessed with the Brief Problem Monitor. Maternal and child social factors were assessed using questionnaire measures and a computerized peer nomination assessment. Our results show that the estimated additive interaction effect for the risk transmission of grandparental and pre- and postnatal parental psychopathology to offspring psychopathology was 23% (95% CI 19; 27). The joint effect of grandparental and parental psychopathology combined with maternal and child social support factors was 13% (95% CI 08; 17)], suggesting that social support factors diminished the intergenerational transmission of psychopathology from (grand)parents (G1 and G2) to offspring (G3). Transmission of psychopathology risk may have long-lasting developmental effects across generations. Social support factors reduced the vulnerability to the effects of psychopathology risk, underscoring the importance of the identification of buffering factors associated with good mental health in adolescents who are at high familial risk.

**Supplementary Information:**

The online version contains supplementary material available at 10.1007/s00787-024-02562-z.

## Introduction

Offspring of parents with psychopathology are at increased risk of developing a wide range of mental health problems, suboptimal educational attainment, poor social outcomes, and a poor quality of the child-parent relationship [1, 2]. Specifically, the transmission of psychopathology from parents to children is a transactional process that unfolds in individuals over time through several social interactions [3], as well as via strong biological mechanisms [4, 5]. However, psychopathology in parents, like any other risk factor, is unlikely to function alone in the intergenerational transmission of psychopathology [6]. Rather, many factors are likely to reduce or strengthen the risk in an interacting manner.

A multitude of studies described that parental psychopathology increases the risk of child psychopathology, with both homotypic continuity (when a psychiatric disorder is predictive of the same condition later on) and heterotypic continuity (when a disorder predicts a different one later in time) commonly reported. [7] A meta-analysis of 33 studies of high-risk families with severe mental illness in parents, suggested that adolescent offspring had a relatively high risk of developing any mental disorder (e.g., psychotic and mood disorders) [8]. For example, offspring of parents with severe mental illness had a 32% higher probability of developing severe mental illness (SMI) (95% CI 24%–42%) by adulthood (age > 20). This risk was more than twice that of control offspring (risk ratio [RR] 2.52; 95% CI 2.08–3.06, *p* < 0.001). Further, studies with a genetically sensitive design suggest that both genetic and environmental transmissions occur [5]. For example, twin studies showed a significant direct environmental transmission from parents to their adolescent offspring for anxiety and neuroticism [4], for depression [9], and for antisocial behavior [10]. Many of these studies have primarily focused on two generations. Only few existing studies adopted a three-generation approach which provided support for the continuity of psychopathology (i.e., anxiety and depression) across multiple generations [11–13]. Weissman et al. found that offspring with two previous generations affected by major depression were at highest risk for major depression suggesting the importance of a family history of depression for children and adolescents [14].

A key point from a developmental psychopathology perspective is to understand individual children’s pathways which support mental health despite being exposed to adversity [15]. Research on family, social, and cognitive factors suggested that having good-quality social relationships outside the family, and having higher self-efficacy beliefs (e.g., perceived ability to overcome problems) [16], are associated with resilience in adolescents [17, 18]. In a study of 9–17 year olds of parents with recurrent depression, Collishaw et al. showed that protective factors (e.g., social relationships and friendships) are associated with less mood and behavior problems at follow-up [16]. To understand the intergenerational continuity of risk and protective factors on the families, these can best be studied with additive interaction analysis as psychopathology risk and social factors may affect children independently.

Several gaps in our understanding remain. First, the periods of exposure assessment (timing of exposure) in prior studies vary, and exposures are rarely assessed repeatedly, whereby we miss knowledge on potential sensitive periods. Second, only little is known about the additive effect of both grandparental and parental psychopathology on offspring psychopathology in the general population. Finally, most studies focus on maternal reports of psychopathology only, whereas paternal psychopathology may capture a different aspect of family functioning or affect adolescents differently.

The present population-based study investigated the intergenerational transmission of liability of psychological problems across three generations (grandparents-G1, parents-G2 and children-G3). Our first aim was to examine the independent main effects of grandparental, parental, and offspring psychopathology (G1 to G2; G1 to G3; and G2 to G3). Second, we investigated an additive interaction effect of grandparental (G1) and parental (G2) psychopathology in association with offspring psychopathology (G3). Third, we examined whether mothers’ perceptions of social support (G2) and adolescents’ perceptions of peer relationships and friendship quality (G3) are associated with the discontinuity of the intergenerational transmission of psychopathology. This design enabled us to study whether the difference in absolute risk between low and high levels of psychopathology transmission from generation to generation differs as a function of resilience factors.

## Methods

### Participants

Our research was embedded in the Generation R Study, a multi-ethnic population-based cohort from fetal life onwards [19]. Briefly, all pregnant women living in Rotterdam, the Netherlands, with an expected delivery date between April 2002 and January 2006 were invited to participate. The current study is a three-generation longitudinal study for which data was used that was collected within the full Generation R cohort and within the smaller Focus Cohort (including Dutch only).

**Fig. 1:**
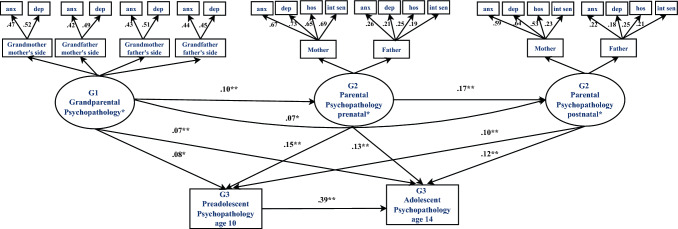
The associations reflecting the intergenerational transmission of psychopathology across three generations: grandparents to parents to children. Structural equation modeling of grandparental and parental psychopathology factors and offspring psychopathology. Numeric values are standardized path regression coefficients of latent factors. The models are adjusted for parental age, parental national origin, education, marital status, smoking and alcohol consumption, child sex and child age. *G1* generation 1 (grandparents), *G2* generation 2 (parents), *G3* generation 3 (offspring). *Latent constructs of grandparental and parental psychopathology. *p < 0.01. **p < 0.001

**Fig. 2:**
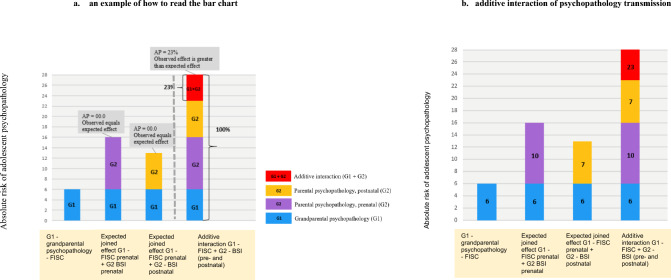
Additive interaction of grandparental and parental psychopathology with offspring psychopathology. The results of the first analysis of additive interaction between grandparental and parental psychopathology. **a** Illustrates an example of how to read the attributable proportion (AP) in the bar charts. **b** Depicts the results of additive interaction between psychopathology across three generations. We found positive additive interaction between grandparental and parental psychopathology. The AP in the last-mentioned ‘bar’ indicates that 23% of the absolute risk of adolescent psychopathology was due to additive interaction. This indicates that 23% of the risk of psychopathology risk among adolescents could be explained by the interaction itself. y-axis: % of the explained variance in absolute risk

Of 8879 women enrolled during pregnancy, we excluded 2230 mothers with missing (G2) psychopathology data, leaving 6649 mothers and 4577 actively participating fathers. Of the remaining families, we excluded those with missing child (G3) psychopathology data on both data points (i.e., self-report at 10 and 14 years, n = 3361 and 3453, respectively), resulting in 4195 children and their parents for G2 to G3 analyses. Information on G1 parents was collected in the 1232 families who constituted the Focus Cohort: parents (G2) were invited for in-depth assessments including an assessment of their parents’ psychiatric profile. There were 816 mothers and 691 fathers who provided information on their parents’ lifetime psychiatric disorders (i.e., the child’s maternal or paternal grandparents) (Supplementary Fig. 1). We randomly excluded 1 child per twin pair (9 in total) and per sibling pair (16 in total). After we excluded an additional 59 mothers without a partner, our main hypothesis was examined in 678 families (mother, father, and child) with information on three generations (G1 to G2 to G3; Fig. 1, 2, and 3d). To address possible bias from differences in sample composition for psychopathology and mediation models, we used analyses of variance tests to compare parental and child characteristics between responders and non-responders at follow-up (Supplementary Method).

**Fig. 3:**
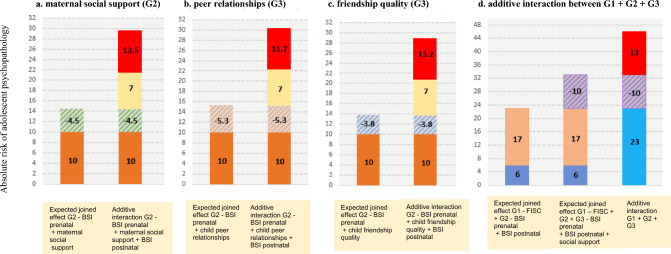
Additive interaction between grandparental and parental psychopathology risk with maternal and child social support. The results of the second analysis of additive interaction between grandparental and parental psychopathology and the combination of social support and friendships. **a** Depicts the results of the analyses of additive interaction between parental psychopathology and maternal social support. **b** Depicts the results of the analyses of additive interaction between parental psychopathology and child peer relationships. **c** Depicts the results of the analyses of additive interaction between parental psychopathology and child friendship quality. **d** Depicts the results of additive interaction between parental psychopathology with parent and child social factors. We found negative additive interaction parental psychopathology and the combinations parent and child social support. The expected joint effect based on summing the independent effects of both grand-parental psychopathology and the combination of maternal and child social support was 13% greater than the observed effect indicated by the AP of 13. *G1* grandparental psychopathology (FISC), *G2* parental psychopathology (BSI). y-axis: % of the explained variance in absolute risk

### Measures

#### Grandparental psychopathology

Lifetime anxiety and depressive disorders of grandparents was assessed when the G2 mothers were 30 weeks pregnant of the G3 children. For this, the Family Informant Schedule Criteria—updated for DSM-IV (FISC) was used [20, 21], which is an interview based on the Family History-Research Diagnostic Criteria (FH-RDC) [22] to assess lifetime psychiatric disorders of relatives. Mothers and fathers completed this interview about their parents during a home interview that was conducted by trained research assistants (Supplementary Material). We used the categorical lifetime psychiatric disorders (categorized as yes/no) as our exposure measures.

#### Parental psychopathology

Mothers and fathers (G2) completed the Brief Symptom Inventory (BSI) to report on their psychiatric symptoms around 20 weeks’ pregnancy and when their child was approximately 3 and 10 years old. In the current study, at each time point we used four subscales, including anxiety, depression, hostility and interpersonal sensitivity. For each assessment we computed the Global Severity Index (GSI) [23], which is the mean score of all items. The Brief Symptom Inventory (BSI) is a widely used instrument to measure self-reported psychopathological symptoms in samples of psychiatric patients and community non-patients [24, 25] (Supplementary Material).

#### Offspring psychopathology

Information on offspring psychopathology at ages 10 and 14 years was obtained using the Brief Problem Monitor (BPM/11-18) [26, 27], a standardized child self-report of problem behaviors. The BPM/11-18 is a validated abbreviated version of the Youth Self-Report (YSR/11-18) [28, 29], and consists of 18 items that yield the three broadband scales Internalizing and Externalizing, and Attention Problems. We used the continuous Total Problems score (the sum of ratings on all items) as our outcome measures (Supplementary Material).

### Social support factors

#### Maternal perceptions of social support

Mothers completed the Social Support List to report on the support they perceived to receive [30]. When the child was 6 months old, mothers completed a 12 item short form questionnaire including three of six subscales, namely, daily-oriented emotional support (e.g., ‘Do others will come to visit you?’), problem oriented emotional support (e.g., ‘Do others offer to help you look after your child(ren)’?), and esteem support (e.g., ‘Do others sometimes emphasize your strong points?’) [31]. For each subscale, we computed the continuous total score, which is the mean score of all items (Supplementary Material).

#### Peer relationships and friendship quality

The PEERS measure is a computerized peer nomination assessment designed for children in the early grades of elementary school (mean child age 8 years) [32]. Children had to imagine going on an exciting school trip and nominated a maximum of six classmates whom they wanted to invite (peer acceptance) and whom they would not want to invite (peer rejection). Using a social network analysis (Statnet package in R), we constructed two networks for each school class based on children’s incoming nominations for peer rejection and acceptance (n = 897) [33].

Researchers visited schools to discuss logistical issues with the directors and teachers, and to tell children about the study. Information on children’s names, dates of birth, and sex were obtained from the school registries. Recent portrait photographs of the participating children (required for peer nomination questions) were either provided by the school or were taken by a researcher during an introduction visit. Before the PEERS measure was administered, the demographic data and photographs were entered into the PEERS assessment program. The procedure children followed when completing the PEERS measure was standardized and a strict protocol was followed at all times (Supplementary Material).

The Friendship Quality was assessed with the Validation and Caring subscale of the Friendship Quality Questionnaire (FQQ), a validated self-report measure of peer acceptance and best friendships [34]. Children (mean age 10 years) completed a short form item questionnaire including one of six subscales, which is designed to assess children’s perceptions of various qualitative aspects of their very best friendship. The FQQ scale consists of 10 items, e.g., ‘Make each other feel important and special’, ‘Has good ideas about games to play”. The continuous FQQ scale was included in the analyses (Supplementary Material).

### Covariates

Maternal and paternal (G2) age was assessed at intake in the study. Parental national origin, education, smoking, alcohol consumption, and marital status were assessed prenatally using self-reported questionnaires. Parents of G2 also reported on grandparental (G1) national origin and education when mothers (G2) were 30 weeks pregnant. Date of birth and sex assigned at birth of the infant (G3) were obtained from community midwife and hospital registries at birth (Supplementary Material).

### Statistical analysis

First, we computed descriptive statistics and the correlations between mother- and father-reported psychopathology scores (G2) at different time points. To examine the transmission of psychopathology varies across generations, the latent constructs based on grandparents’ (G1) and parents’ psychopathology (G2) reported by mothers and fathers were constructed (Supplementary Material). We then used structural equation modeling (SEM) to test the associations of measures of grandparental and parental psychopathology latent constructs (measured prenatally and at child ages 3 and 10) in relation to offspring psychopathology (self-report measured at ages 10 and 14) (Fig. 1). We ran the models adjusting for all previously mentioned confounders.

Including both maternal and paternal reports in the same SEM model addresses variance between informants and thus was our model of choice. However, we also present an additive interaction model to better understand whether the difference in absolute risk between low and high levels of psychopathology among children differs as a function of psychopathology risk of grandparents and parents. This model quantifies inferred effects of both parental and grandparental psychopathology, and shows that additive effects can increase psychopathology risk in offspring by combining parental and grandparental psychopathology. We estimated the patterns of risk for joint exposure to different combinations of risk factors for grandparents’ and parents’ heterotypic psychopathology, and examine whether these patterns are consistent with additive relationships. Our a priori expectation was that in case of joint exposure to risk factors (grandparents’ and parents’ psychopathology), the risk of psychopathology would be greater than that expected under an additive model for all psychopathology combinations studied. Under an additive model, the null hypothesis is that risks for each exposure combine additively (for example, Risk_A and B_ = Risk_A only_ + Risk_B only_–Risk_Neither A nor B_). If the risk when exposed to both A and B (e.g., joint effect of grandparents’ and parents’ psychopathology) is greater than this formula, or less than additive, there will be statistical interaction under an additive model (Fig. 2). This model might be more effective than multiplicative interaction in capturing the effect of exposures, such as grandparental and parental psychopathology, or social factors, that may act either independently or synergistically on the same outcome (offspring psychopathology) [35]. We calculated the absolute risk of offspring psychopathology for each combination of higher levels of grandparental and parental psychopathology.

In the second interaction analysis, to test to what degree risk and protective factors are additive (linearly increasing) or redundant (not adding to each other), five exposure states were used by the combination of each risk and protective factor (Fig. 3). That is, we tested additive interaction between joint effects of grandparental and parental psychopathology with different combinations of protective factors, including maternal perceptions of social support, child peer relationships and friendship quality, on offspring psychopathology. In this analysis, we calculated the attributed risk in adolescents that were exposed to both grandparental and parental psychopathology and each of the different combinations of maternal and child protective factors.

Lastly, the main effects of the associations between grandparents’ (G1) and parents’ psychopathology (G2) as assessed at each time point (without using a latent construct) with offspring psychopathology (G3), were determined with separate linear regressions.

To address missing values for all variables, we used full information maximum likelihood (FIML) [36]. Supplementary Methods describe attrition from baseline and selection of our analytic samples. All analyses were performed using SAS 9.4 software (Supplementary Material).

## Results

**Fig. 4:**
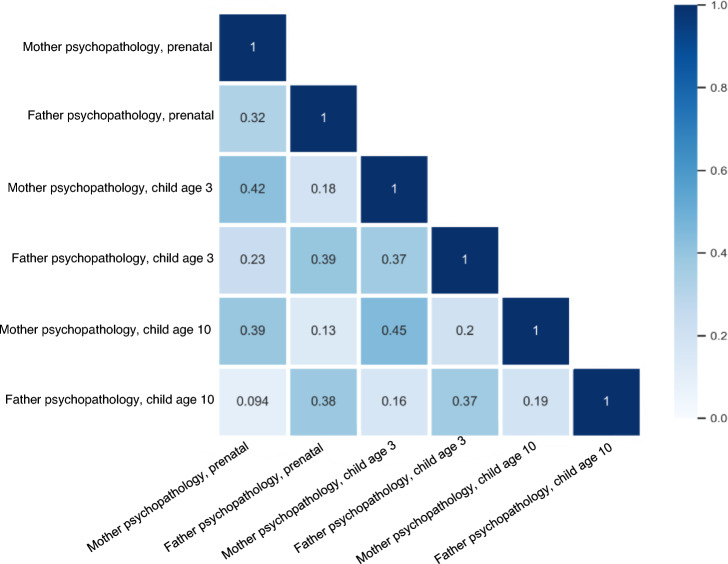
Correlations between maternal and paternal psychopathology

The descriptive sample characteristics of sociodemographic factors are shown in Table 1. The mean age of the adolescents at the latest outcome assessment was 13.6 years, and 49.8% of the adolescents were boys. In total, 37.2% of mothers and 29.5% of fathers had a Non-Western origin. The correlations of psychopathology across generations are shown in Fig. 4.

**Table 1:** Sociodemographic characteristics

	Grandparents		Parents		Offspring
	Mother	Father	Mother	Father	
Age, M (SD)			30.1 (5.1)	32.9 (5.6)	13.6 (.39)
Child sex, (% girl)					50.2
Parental national origin					
Dutch (%)	41.4	59.1	53.8	63.5	
Other Western (%)	7.3	6.5	8.9	7.1	
Non-Western (%)	51.3	34.4	37.2	29.5	
Education level					
High (%)	40.1	46.2	45.8	51.0	
Middle (%)	37.4	24.0	30.3	26.6	
Low (%)	22.5	29.8	23.9	22.4	
Alcohol use during pregnancy					
No consumption during pregnancy (%)			37.4		
Until pregnancy recognized (%)			13.8		
Continued occasionally (%)			38.4		
Continued frequently (%)			10.4		
Smoking during pregnancy					
No smoking during pregnancy (%)			73.1		
Until pregnancy recognized (%)			8.4		
Continued during pregnancy (%)			18.5		
Single parenthood during pregnancy (%)			13.2		
Grandparents of mothers’ side (%)					
Lifetime anxiety disorder	10.8				
Lifetime depressive disorder	22.3				
Grandparents of fathers’ side (%)					
Lifetime anxiety disorder		10.1			
Lifetime depressive disorder		16.5			
Parental psychopathology, M (SD)					
Prenatal			0.30 (.38)	0.14 (.23)	
At age 3 years			0.15 (.25)	0.13 (.22)	
At age 10 years			0.22 (.31)	0.16 (.22)	
Offspring psychopathology, age 10, M (SD)					7.55 (5.0)
Offspring psychopathology, age 14, M (SD)					31.3 (18.9)
Maternal social support, age 6 months, M (SD)			2.73 (0.42)		
Peer relationships, age 8, M (SD)					0.16 (0.8)
Friendship quality, age 10, M (SD)					2.40 (.33)

Numbers denotes children included in one or more analyses. Values are frequencies for categorical and means and standard deviations (M ± SD)

Next, we tested whether psychopathology risk varies across generations (Fig. 1), and found that grandparental psychopathology (G1) was associated with parental psychopathology (G2) measured during pregnancy (β = 0.10 SE = 0.04; *p* = 0.001) and in early childhood (β = 0.17 SE = 0.05; p = 0.004), as well as with preadolescent (G3) (β = 0.08 SE = 0.03; *p* = 0.010) and adolescent psychopathology (β = 0.07 SE = 0.02; *p* = 0.022). That is, latent constructs of grandmother’s and grandfather’s psychopathology (e.g., anxiety and depression disorders) were associated with psychopathology of their children and grandchildren. The magnitude of these associations was modest, accounting for a small variance in children’s psychopathology. Prenatal parental psychopathology (G2) was associated with more psychopathology in children at age 10 (β = 0.15 SE = 0.07; *p* = 0.001), and 14 (β = 0.13 SE = 0.05; *p* = 0.001). Similar association patterns over time were observed between postnatal parental psychopathology (G2) and offspring psychopathology scores at ages 10 (β = 0.10 SE = 0.03; *p* = 0.001), and 14 (β = 0.12 SE = 0.04; *p* = 0.001), with children exposed to higher parental psychopathology scores having higher preadolescent and adolescent psychopathology scores.

Figure 2 shows the results of the additive interaction effect on how exposure to a combination of risk factors—i.e., the latent constructs of grandparents’ and parents’ heterotypic psychopathology—is associated with offspring psychopathology. We observed that significant additive interactions among heterotypic psychopathology occur for both grandparental and repeatedly measured pre- and postnatal parental psychopathology in association with offspring psychopathology. The attributed risk (AR) was 23%, as indicated by the attributed proportion of the psychopathology risk exposure of 23, 95% CI: 19; 27, (Fig. 2b). That is, the joint relative risks of both grandparental and parental psychopathology with offspring psychopathology are for the most part significantly more than the product of the relative risks associated with each parent or grandparent psychopathology risk alone.

Next, we found an additive effect between parental psychopathology (G2) over time and maternal perceptions of social support (G2), suggesting that maternal social support reduces adolescents’ psychopathology by 4.5% [indicated by the AR of -4.5 (95% CI: -7.3; -2.4)] (Fig. 3a). We also found a similar additive interaction effect between parental psychopathology (G2) and child peer relationships (G3) reducing the associations of offspring psychopathology by 5.3% [indicated by the AR of −5.3 (95% CI: −8.5; −2.6)]. Further, child friendship quality (G3) reduces the association of parental psychopathology (G2) on adolescents’ psychopathology (G3) by 3.8% [indicated by the AR of −3.8 (95% CI: −5.1; −1.7)] (Fig. 3b, c). This pattern suggests the presence of additive interactions between psychopathology and social support factors; that is, the attributed risk of grandparent s’ and parent s’ psychopathology in relation to offspring psychopathology decreases as the number of social support factors increases.

As illustrated in Fig. 3d, the joint effect between grandparental and parental psychopathology and the combinations of the three maternal and child social support factors was 13% [indicated by the AR of 0.13 (95% CI: 08; 17)], suggesting that social support factors, both separately and in combination, diminished the associations of grandparents’ and parents’ psychopathology (G1 and G2) with offspring psychopathology (G3).

Lastly, the associations between grandparents’ and parents’ psychopathology as assessed at each time point with offspring psychopathology are shown in the Supplementary Table 1 and 2.

## Discussion

This population-based cohort study from fetal life onwards suggests that previous generations’ experiences of liability for emotional and behavioral problems are reflected in the next generation’s psychopathology. In particular, we found that grandparental psychopathology reflected continuity to the next two generations. The grandparental and parental psychopathology continuity was mainly defined by additive interaction effects, so that psychopathology of grandparents increased 6% risk in parental psychopathology. The overall risk of both grandparental and parental psychopathology transmission to adolescents was 23%. We found that maternal perceptions of social support, child peer relationships and friendship quality reduced risks from 23 to 13% in the context of psychopathology in previous generations. Overall, our findings suggest that parent and child social support factors buffered the effect of psychopathology experiences in an intergenerational continuity.

The present study extends the available psychopathology literature by including both parents and underscores the multiple pathways by which not only parents’ psychopathology during prenatal and early child life but also grandparents’ psychopathology may be associated with child psychopathology. We found evidence for continuity of lifetime psychopathology from G1 grandparents to their children G2, and that, a history of lifetime psychopathology in G1 was also directly associated with more psychopathology in grandchildren (G3). However, the associations (effect sizes) were small in magnitude, for example, G1 psychopathology explained a relatively limited amount of variance in G3 psychopathology. Our results are consistent with the extant literature, showing that children with both a parent and a grandparent with depression had higher rates of psychiatric disorders compared to children with only a parent with depression, independent of sex, race/ethnicity, and socioeconomic status [37]. However, mechanisms underlying broad psychopathology (heterotypic patterns) are still under debate. Indeed, some heterotypic patterns disappear when comorbidities (consisting of homotypic patterns) are considered. The associations between disorders may also be mediated by comorbidity. These factors suggest that the actual prevalence of heterotypic continuity is inflated [38]. Other researchers have found that heterotypic continuity persists even when controlling for comorbidity [39, 40]. For example, a longitudinal study by Lahey et al. 2014 suggests that heterotypic patterns persist even after correcting for homotypic ones [41]. Direct associations of broad psychopathology (heterotypic patterns) from G1 to G2 and from G2 to G3 may represent social modeling, inherited vulnerabilities, and continuities in detrimental environments that are not influenced by contextual stressors examined in the current study.

Further, in addition to the relative risk of low versus high levels of psychopathology that varies across generations, the difference in absolute risk between low and high levels of psychopathology differs as a function of psychopathology risk of grandparents and parents for children experiencing only one parent or neither with psychopathology (as depicted in Fig. 2). This model represents continuity to heterotypic psychopathology that may independently account for the intergenerational transmission of psychopathology across generations.

Our results are consistent with the dynamic developmental systems (DDS) model [42] that has emphasized that both parents’ risk characteristics and interpersonal interactions directly contribute to the family risk environment and therefore contribute to offspring adjustment. This model provides a comprehensive framework to explain the intergenerational transmission of psychopathology through various mechanisms, including genetic transmission, exposure to maladaptive parental interactions, and contextual stressors. First, it is likely that parental psychopathology reflects biological vulnerability to offspring psychopathology. Biological vulnerability can be based on shared genetically characteristics [43], which may increase offspring susceptibility to develop psychopathology. There is some evidence showing that shared genes may affect child and parent behaviour directly, resulting in similarities in behaviour across generations [13]. That is, genetic makeup of grandparents of G1 and parents (G2) could have an independent impact on offspring of G3. Associations representing the intergenerational transmission of psychopathology were more pronounced for maternal psychopathology during pregnancy compared to early childhood, which seemingly implies pregnancy specific effects in which, for example, maternal psychopathology may lead to direct physiological changes that could affect fetal development. Another mechanism is exposure to parents’ maladaptive interactions, behavior and cognitions, which can lead to dysfunctional modeling and high family stress. Furthermore, contextual stressors, such as family disruption that may arise due to parents’ psychopathology, could be related to the development of child psychopathology [44]. For example, parents with psychopathological problems may have less time to facilitate children’s social activities. As such, children of parents with psychopathological symptoms may be more likely to experience a less optimal environment which underlies the relation with brain developmental. Although we cannot conclude which of these mechanisms contributed most to these associations, results of our study help guide the mechanistic understanding. Moreover, we carefully controlled for contextual stressors.

There is some evidence that psychosocial support [45, 46], including pair bonding, friendship, and family bonds moderate both genetic and environmental vulnerabilities and confer resilience to stress and adversity [47–51]. Consistent with the study hypotheses, G2 maternal perceptions of social support shortly after child birth were associated with lower levels of psychopathology in G3 offspring. This result suggests that social support in one generation can have a cascade of positive effects across subsequent generations. Finding that social support in early childhood is related to less psychopathology in offspring offers powerful support for the potential value of prevention efforts.

Furthermore, we observed that G3 child peer relationships and friendship quality contributed to fewer psychopathology symptoms among G3 children. Each association has been supported in prior studies, but much less considered is the additive effect of constructs (social support factors) simultaneously. Moreover, these factors were related but nonoverlapping constructs in adolescence. That is, children with more friends and with more positive relationships with friends are less vulnerable to parental psychopathology than those having less friends or less positive relationships. Together, these findings suggest that the experience of having positive, supportive relationships may buffer against the intergenerational transmission of psychopathology to some extent. The current study advances the DDS [42] theory by evaluating maternal and offspring social support factors (maternal social support, peer relationships, and friendship quality) as a mechanism through which social support factors maintain across generations. It is important to note that we simultaneously model psychopathology across generations and social factor pathways. This approach stringently tests whether the latter offers a meaningful explanatory mechanism of social support transmission or is simply an artifact of powerful continuities in problem behaviors and psychopathology across generations. Moreover, the inclusion of fathers’ psychopathology in the study model strengthens general conclusions that can be drawn regarding intergenerational processes. Although, psychopathology factors reflect the common variance across mother- and father-reported psychopathology, the clear association found for paternal-reported psychopathology suggests that direct paternal changes may also underlie the findings.

The results also suggest that multiple protective factors are required, as less psychopathology was reported only if mothers and adolescents reported a combination of multiple protective factors across home, school, and peers. This is in line with the multi-system theory of resilience suggesting that efforts to target single protective factors in isolation for at-risk adolescents will probably not be sufficient to prevent the development of symptoms of psychopathology [18, 52]. This is further supported by the notion that resilience is not simply the absence of psychopathology but a construct that reflects dynamic adaptation to adversity that can change over time [53]. As children develop, their capacity to impact characteristics of their environments increases. For example, given that parents (and their behaviors) represent a central component of this environment, children’s ability to engage in meaningful interactions with parents will be greater than of infants, or might be bidirectional [54]. It is also possible that both parent and child effects will weaken over time, because children become less dependent on parents over time and more influenced by peers and other social factors [55, 56], although child- and parent-driven processes that combine to shape the home environment will remain relevant.

This study has several limitations. First, parents (G2) and children (G3) reported on their own psychopathology, whereas grandparental psychopathology disorder constructs (G1) were obtained from maternal and paternal reports of G2. Parental reports of G2 about their own parents’ psychiatric disorders can either inflate or deflate the observed associations. However, as only definite lifetime psychiatric disorders were considered (categorized as absence or presence of a disorder), the risk of inflation is minimized. Second, this study has a population-based design, but the relative homogeneity of the population limits its generalizability. Third, it is likely that the associations of parents to offspring psychopathology are not unidirectional, but might also reflect some bi-directionality that we did not capture. The strength of our study lies in its longitudinal design investigating the psychosocial mechanisms underlying the intergenerational transmission of psychopathology. Furthermore, the large number of participants and broad spectrum of measured covariates enabled us to adjust for multiple confounders. The methodology used in this study enabled us to assess an additive interaction effect testing psychopathology and social support factors jointly in one model.

The identification of factors associated with good mental health in adolescents who are at high familial risk has important implications for treatment and prevention. First, our findings suggest that treatment of parents is a priority but might be insufficient as a single factor to prevent the risk of psychopathology in offspring. Moreover, in both mothers and their offspring social support factors contributed to resilience, which suggests that multiple protective factors are required to reduce the influence of strong intergenerational risks. Thus, there are potential modifiable targets for preventive interventions that might help interrupt the intergenerational transmission of risk for psychopathology. Specifically, programs incorporating elements of cognitive behavioral therapy focused on improving social support are likely to be important for youth to prevent the intergenerational transmission of psychopathology [18]. Persistent stress and insufficient coping strategies, in which sufficient social support can play an important role, have negative consequences for mental and physical health especially in offspring at high familial risk. Educating parents and their offspring in the skills needed to sustainably improve stress-buffering coping, like social support, could contribute to the prevention of psychopathology. However, one of the main conclusions from the research on social support and psychopathology relates to how complicated the provision of social support in everyday life is, yet how beneficial it is for psychological functioning. However, it is crucial to move beyond a general expectation that providing social support in itself will yield many benefits, because it cannot address the complex needs of individuals. Programs must therefore recognize that social support is multifaceted; individuals have diverse needs, expectations, and personal and cultural backgrounds and networks [57]. Awareness regarding the importance of social support as an essential component of prevention and therapeutic programs, is still limited and needs more attention both in research and in clinical practice.

In conclusion, our findings show that grandparental psychopathology (G1) was associated with changes across parental psychopathology (G2) by increasing levels of offspring emotional and behavioral problems (G3). This suggests that transmission of psychopathology risk may have long-lasting developmental consequences across generations. Moreover, parent and child social support factors buffered the effect of psychopathology experiences, suggesting that several protective factors together are likely to promote adolescents’ mental health resilience. Further research should identify the child’s contribution to parental psychopathology, as a potential mechanism for the intergenerational continuity of psychopathology.

## Supplementary Information

Below is the link to the electronic supplementary material.

Supplementary file1 (DOCX 181 KB)

## Data Availability

Data can be obtained upon request. Requests should be directed to the towards the management team of the Generation R Study (secretariaat.genr@erasmusmc.nl), which has a protocol of approving data requests. Because of restrictions based on privacy regulations and informed consent of participants, data cannot be made freely available in a public repository.
