# Supplementary material for: Intergenerational transmission of psychopathology across three generations: the role of social support
Source: Eur Child Adolesc Psychiatry. 2024 Aug 17;34(4):1329–40. doi: 10.1007/s00787-024-02562-z (PMC12000276; doi:10.1007/s00787-024-02562-z)
Supplement: Supplementary file 1 — Supplementary file1 (DOCX 181 KB) [file 787_2024_2562_MOESM1_ESM.docx]

**Supplementary material**

**Methods**

**Measures**

**Grandparental psychopathology**

The FH-RDC showed good inter-rater reliabilities ranging from kappa = 0.72 for depression to 0.75 for anxiety [1]. Mothers and fathers were interviewed separately about their parents’ psychiatric disorders, with the partner not present in the same room at the time of the interview. Three levels of confidence for the diagnoses are generated; ‘definite’, ‘probable’ or ‘absent’. For the current study, we considered only ‘definite’ diagnoses as positive diagnoses. Groups of diagnoses included in the present study were: 1) lifetime history of an anxiety disorder, consisting of generalized anxiety disorder, obsessive-compulsive disorder, panic disorder, agoraphobia, social phobia, specific phobia or posttraumatic stress disorder and 2) lifetime history of a (unipolar) depressive disorder consisting of a major depressive episode or dysthymia. Internal consistencies (Cronbach’s alpha) ranged from 0.71 to 0.78.

**Parental psychopathology**

In the current study, at each time point we used four subscales including anxiety, depression, hostility and interpersonal sensitivity. For each assessment we computed the Global Severity Index (GSI) [2], which is the mean score of all items. The BSI is a validated self-report questionnaire with 53 items to be answered on a five-point scale, ranging from 0 = ‘not at all’ to 4 = ‘extremely’ [3, 4] High validity and reliability have been reported for the Dutch translation [2]. Internal consistencies (Cronbach’s alpha) ranged from 0.75 to 0.81 across the different waves.

**Offspring psychopathology**

Similar to the BPM and YSR, the Child Behavior Checklist (CBCL/6-18) was used to obtain a parent-report of child problems at ages 10 and 14. Good reliability and validity have been reported for the CBCL/6-18 and YSR and BPM.[5] Each item is scored on a three point rating scale 0 = 'not true', 1 = 'somewhat or sometimes true', and 2 = 'very true or often true', based on the preceding two months. In the current study, internal consistencies (Cronbach’s alpha) ranged from 0.70 to 0.74.

**Maternal perceptions of social support**

Mothers completed the Social Support List to report on the support they perceived to receive. The validated Social Support List measures several dimensions of support which were commonly reported in a Dutch sample.[6] The items were answered on a four-point scale, ranging from 1 = ‘I miss it’ to 4 = ‘Too much support’.[7]

**The friendship quality**

The FQQ scale consists of 10 items, e.g., ‘Make each other feel important and special’, ‘Has good ideas about games to play”, ‘Tells me I am good at things”, to be answered on a five-point scale, ranging from 0 = ‘to a little true’ to 4 = ‘to really true’, indicating how true a particular quality is of their relationship with a specific friend [8].

**Peer relationships**

In the peer acceptance network, high centrality scores indicate that a child is highly liked or popular, whereas low centrality scores reflect a more peripheral position. The degree of reciprocity (i.e. the proportion of mutually returned nominations) reflects how balanced a child's peer relationships are in a given network and provide an indication of mutual antipathies or “enemies” (peer rejection network) and “friendships” (peer acceptance network). In the analyses, we used the networks of peer acceptance and peer rejection.

The teachers of the participating classes needed to make no substantial time investment, as all testing was done by the researchers [9]. Before administration of the measure started, a researcher gave children instructions about the PEERS Measure. After the general introduction, children were seated at computers, each at a sufficient distance to ensure privacy. Once the task started, children heard a short introduction and instructions via a headset. The assessment began with a self-identification task to check whether a child could recognize him or herself and his or her classmates in the photos. Then, to familiarize children with the nomination technique, two exercise questions followed during which children were asked to nominate an animal they liked most and an animal they liked least.

Next, children were asked to nominate their classmates. This part of the PEERS Measure started with questions about peer acceptance and rejection. The children were told to imagine that they were going on an exciting school trip and could nominate not only children they would like to take with them (peer acceptance) but also those they would rather not take (peer rejection). To answer these questions, they should click on the photos of the classmates, which were displayed in random order. Children could nominate classmates by clicking on their photographs. The number of nominations was restricted to six for peer-acceptance and peer-rejection questions.

We chose to computerize the task, first for reasons of efficiency and standardization and also to avoid situational effects related to an interviewer's possible influence on a child. Asking open questions was not feasible, as children were intended to complete the task independently. Because it was also important for the nominations to be registered automatically and to be restricted solely to the participating children, the children were presented with the pictures of their participating in the study classmates and were asked to click on the relevant photos to nominate children who behaved toward them in the way described. If a child wished to nominate a classmate who was not participating in the study, he or she could click on a special “dummy” picture with no photograph.

A trained research assistant supervised children completing the PEERS assignment and was available for questions and help at all times. The average time taken to complete the assignment was 7.6 min (SD = 1.9 min). Anonymous ID numbers for all the participating children were generated by the PEERS Measure program. A data set containing coded data were created automatically after the PEERS Measure was conducted in each class [9].

**Covariates**

Parental ethnicity was based on country of birth of mothers and fathers, as assessed in questionnaires during pregnancy, then categorized into Dutch, non-Western and other Western national origin [10]. Parental education was classified in three levels: ‘low’ (maximum of three years general secondary school), ‘medium’ (>3 years general secondary school; intermediate vocational training), and ‘high’ (Bachelor’s degree or higher academic education). Parents of G2 also reported on grandparental (G1) national origin (Dutch, non-Western, and other Western), and education (‘low’, ‘medium’, and ‘high’) when mothers (G2) were 30 weeks pregnant. Information about smoking (three categories: no smoking during pregnancy, smoked until pregnancy recognized, and continued smoking during pregnancy), alcohol intake during pregnancy (four categories: no alcohol consumption during pregnancy; alcohol consumption until pregnancy recognized; continued occasionally during pregnancy (<1 glass/week); and continued frequently during pregnancy (1+ glass/week)), was prenatally assessed by questionnaires. Marital status (two categories: ‘married/living together’ and ‘separated/divorce’) was assessed prenatally using self-report questionnaires. Date of birth and sex assigned at birth of the infant (G3) were obtained from community midwife and hospital registries at birth.

**Statistical analysis**

*Full maximum likelihood estimation:* FIML avoids uncertainties from estimating data and provides unbiased estimates of missing parameters in large sample size while retaining natural variability in missing data, assuming missing data at random (MCAR) [11]. Thus, each participant contributes to the data they have available at each time point to the likelihood function and no participants are removed from analyses through list-wise deletion. In addition, we compared our findings with and without FIML procedures (i.e., list-wise deletion was employed) and found no evidence that our estimates were biased by the missing data.

*Latent constructs:* For each grandparent (G1), a standardized latent construct of psychopathology was constructed based on the two assessed psychopathology domains (i.e., anxiety and depressive disorders). Using these, a latent factor was constructed for each grandmother-grandfather dyad (from either mothers’ or fathers’ side). Then, latent constructs were made for maternal and paternal reports of psychopathology (G2) during pregnancy and early childhood (including four psychopathology domains: anxiety, depression, hostility, and interpersonal sensitivity, see Figure 1). The latent constructs showed a good model fit as judged with the comparative fit index (CFI, acceptable fit ≥ .90) [12]. The latent constructs were allowed to correlate, and were estimated with the robust maximum likelihood estimator using standardized latent variables. The association between the latent construct of parental psychopathology and offspring psychopathology captures covariation across raters, or the extent to which a given dimension is reflected across parents (i.e., between-rater dimension factor).

***Additive interaction analysis Code***

Below is an example of the additive interaction code [13] used to examine whether the difference in absolute risk between low and high levels of psychopathology differs as a function of psychopathology risk of grandparents and parents compared to children experiencing only one or neither in “SAS” version 9.4.

*proc logistic descending data = mydata outest = myoutput covout;*

*model d = g e g*e p1 p2 p3;*

*run;*

**Attrition analysis in the Generation R Study**

Children who were lost to follow-up (N=1,776) reported slightly lower rates of psychopathology at age 10 years (χ2 = 423.21, p = 0.030) and at age 14 years (χ2 = 346.40, p = 0.001), more often had parents of Non-Western national origin (χ2 = 165.75, p <0.001), had parents with lower educational attainment (χ2 = 132.58, p <0.001), used more alcohol during pregnancy (χ2 = 98.14, p <0.001), and continued smoking during pregnancy (χ2 = 106.72, p <0.001), than adolescents who were retained at follow-up (N = 4,195).

**Supplementary Figure 1. Flowchart of the study sample**


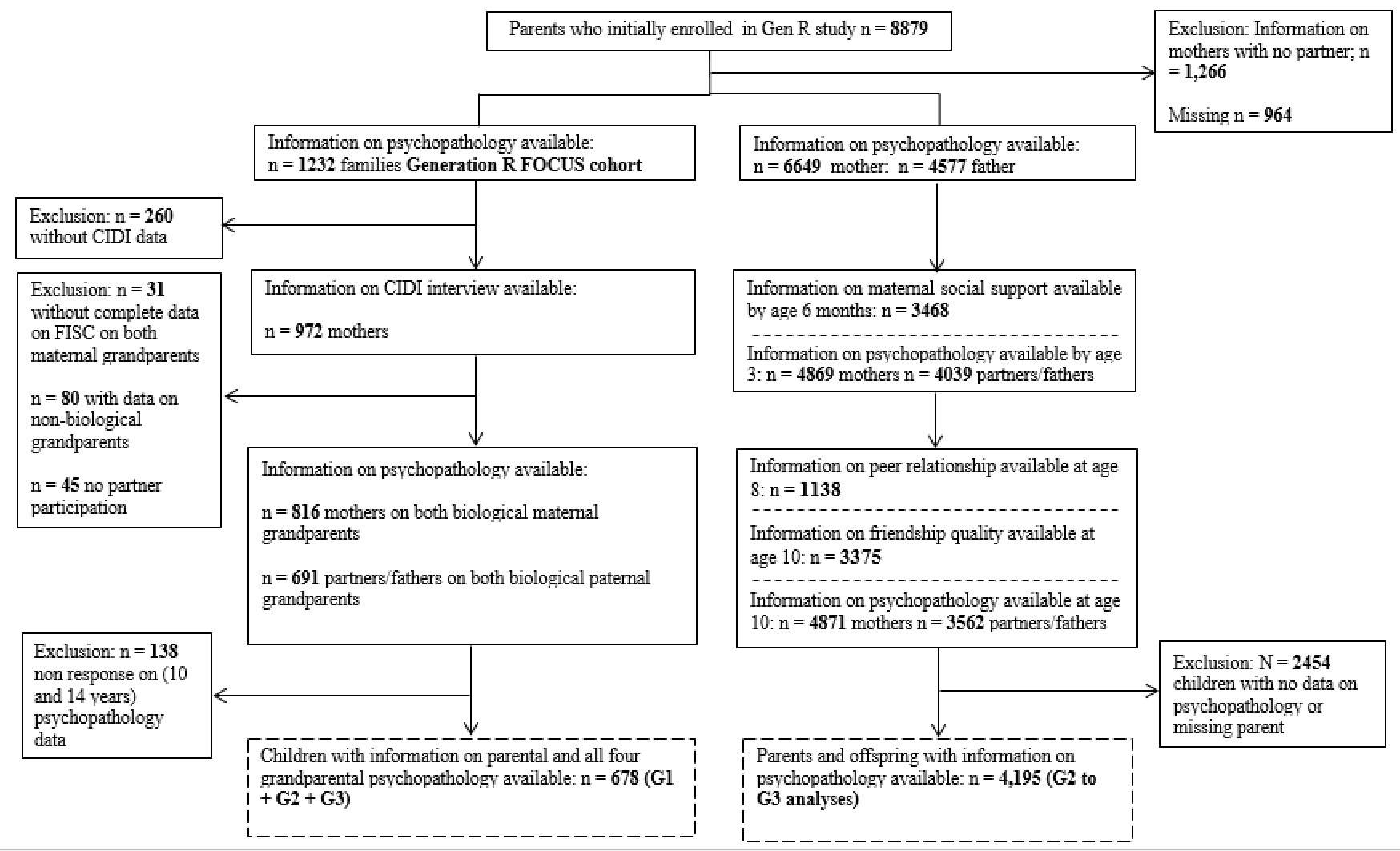


**Supplementary Table 1. The associations of grandparental psychopathology with parental and offspring psychopathology**

|  | **Parental psychopathology,**  **prenatal (G2)** | | **Parental psychopathology,**  **child age 3 (G2)** | | **Parental psychopathology,**  **child age 10 (G2)** | | **Preadolescent psychopathology**  **self-report, age 10 (G3)** | | **Adolescent psychopathology**  **self-report, age 14 (G3)** | |
| --- | --- | --- | --- | --- | --- | --- | --- | --- | --- | --- |
|  | β (95% CI) | *p* | β (95% CI) | *p* | β (95% CI) | *p* | β (95% CI) | *p* | β (95% CI) | *p* |
|  |  |  |  |  | **Mothers** |  |  |  |  |  |
| **Grandparental psychiatric disorders, mother side (G1)** |  |  |  |  |  |  |  |  |  |  |
| Model 1 | 0.45 (0.27 - 0.59) | 0.005 | 0.31 (0.15 - 0.53) | 0.010 | 0.23 (0.14 - 0.45) | 0.017 | 0.20 (0.12 - 0.34) | 0.003 | 0.16 (0.08 - 0.36) | 0.010 |
| Model 2 | 0.28 (0.19 - 0.48) | 0.010 | 0.30 (0.15 - 0.52) | 0.016 | 0.20 (0.14 - 0.41) | 0.020 | 0.16 (0.08 - 0.31) | 0.008 | 0.13 (0.07 - 0.39) | 0.034 |
|  | **Fathers** | | | | | | | | | |
| **Grandparental psychiatric disorders, father side (G1)** |  |  |  |  |  |  |  |  |  |  |
| Model 1 | 0.52 (0.44 - 0.71) | 0.001 | 0.45 (0.31 - 0.64) | 0.001 | 0.36 (0.20 - 0.53) | 0.009 | 0.30 (0.19 - 0.48) | 0.001 | 0.27 (0.18 - 0.27) | 0.026 |
| Model 2 | 0.48 (0.40 - 0.66) | 0.003 | 0.43 (0.30 - 0.64) | 0.005 | 0.34 (0.18 - 0.49) | 0.018 | 0.29 (0.18 - 0.48) | 0.002 | 0.25 (0.14 - 0.33) | 0.029 |

Note: Linear regression analysis between grandparental psychopathology with parental and offspring psychopathology across three generations. Betas are averaged from 20 imputed

datasets. Model 1 is adjusted for child sex and age. Model 2 is additionally adjusted for maternal or paternal age, national origin, education, and marital status, smoking and alcohol

consumption.

**Supplementary Table 2. The associations of parental and offspring psychopathology**

|  | **Preadolescent psychopathology,**  **self-report**  **(n = 2,742)** | | **Preadolescent psychopathology,**  **mother (n = 2,962) and father (n = 2,356) reports** | | **Adolescent psychopathology,**  **self-report**  **(n = 3,470)** | | **Adolescent psychopathology,**  **mother reports**  **(n = 3,400)** | |
| --- | --- | --- | --- | --- | --- | --- | --- | --- |
|  | β (95% CI) | *p* | β (95% CI) | *p* | β (95% CI) | *p* | β (95% CI) | *p* |
| **Maternal psychopathology, prenatal** |  |  |  |  |  |  |  |  |
| Model 1 | 0.31 (0.17 - 0.44) | <0.001 | 0.79 (0.67 - 0.91) | <0.001 | 0.39 (0.29 - 0.51) | <0.001 | 0.69 (0.58 – 0.79) | <0.001 |
| Model 2 | 0.28 (0.14 - 0.42) | <0.001 | 0.77 (0.65 - 0.91) | <0.001 | 0.42 (0.30 - 0.53) | <0.001 | 0.67 (0.55 - 0.77) | <0.001 |
| **Maternal psychopathology, child age 3** |  |  |  |  |  |  |  |  |
| Model 1 | 0.58 (0.39 - 0.76) | <0.001 | 1.08 (0.92 - 1.26) | 0 .001 | 0.51 (0.34 – 0.67) | <0.001 | 1.00 (0.84 - 1.16) | <0.001 |
| Model 2 | 0.56 (0.38 - 0.75) | <0.001 | 1.05 (0.88 - 1.22) | <0.001 | 0.50 (0.34 - 0.68) | <0.001 | 0.97 (0.82 - 1.14) | <0.001 |
| **Maternal psychopathology, child age 10** |  |  |  |  |  |  |  |  |
| Model 1 | 0.52 (0.39 - 0.65) | <0.001 | 1.18 (1.06 - 1.29) | <0.001 | 0.45 (0.32 - 0.57) | <0.001 | 0.88 (0.77 - 1.01) | <0.001 |
| Model 2 | 0.51 (0.37 - 0.64) | <0.001 | 1.15 (1.04 - 1.28) | <0.001 | 0.44 (0.31 - 0.57) | <0.001 | 0.85 (0.73 - 0.95) | <0.001 |
|  |  |  |  |  |  |  |  |  |
| **Paternal psychopathology, prenatal** |  |  |  |  |  |  |  |  |
| Model 1 | 0.74 (0.52 - 0.96) | <0.001 | 1.37 (1.20 - 1.55) | <0.001 | 0.58 (0.39 - 0.77) | <0.0001 | 0.67 (0.48 - 0.87) | <0.001 |
| Model 2 | 0.69 (0.47 - 0.91) | <0.001 | 1.13 (0.91 - 1.37) | <0.001 | 0.57 (0.39 - 0.77) | <0.001 | 0.65 (0.47 – 0.82) | <0.001 |
| **Paternal psychopathology, child age 3** |  |  |  |  |  |  |  |  |
| Model 1 | 0.59 (0.39 - 0.76) | <0.001 | 1.20 (1.01 - 1.47) | 0.001 | 0.59 (0.39 - 0.74) | <0.001 | 0.90 (0.72 - 1.01) | <0.001 |
| Model 2 | 0.58 (0.37 - 0.81) | <0.001 | 1.20 (0.98 - 1.44) | 0.001 | 0.58 (0.38 - 0.77) | <0.001 | 0.89 (0.70 - 1.07) | <0.001 |
| **Paternal psychopathology, child age 10** |  |  |  |  |  |  |  |  |
| Model 1 | 0.50 (0.31 - 0.68) | <0.001 | 1.37 (1.20 - 1.55) | <0.001 | 0.61 (0.44 - 0.79) | <0.001 | 0.65 (0.48 - 0.82) | <0.001 |
| Model 2 | 0.49 (0.31 - 0.67) | <0.001 | 0.32 (0.12 - 0.51) | 0.001 | 0.60 (0.44 - 0.78) | <0.001 | 0.64 (0.47 - 0.82) | <0.001 |

Note: Linear regression analysis of parental and offspring psychopathology across two generations. Betas are averaged from 20 imputed datasets. Model 1 is adjusted for child

sex and age. Model 2 is additionally adjusted for maternal or paternal age, national origin, education, and marital status, smoking and alcohol consumption.

**References:**

[1] U. Ptok, C. Seeher, F. Jessen, A. Papassotiropoulos, and R. Heun, "Inter-rater reliability of family history information on psychiatric disorders in relatives," *Eur Arch Psychiatry Clin Neurosci,* vol. 251, no. 6, pp. 279-83, Dec 2001.

[2] De Beurs and F. Zitman, "De Brief Symptom Inventory (BSI)," *De betrouwbaarheid en validiteit van een handzaam alternatief voor de SCL-90. Leiden: Leids universitair medisch centrum,* 2005.

[3] de Beurs, "Brief symptom inventory," *Handleiding. Leiden (Netherlands): Pits Publishers,* 2004.

[4] L. R. Derogatis and N. Melisaratos, "The Brief Symptom Inventory: an introductory report," (in eng), *Psychol Med,* vol. 13, no. 3, pp. 595-605, Aug 1983. [Online]. Available: <http://www.ncbi.nlm.nih.gov/pubmed/6622612>.

[5] T. M. Achenbach, S. H. McConaughy, M. Y. Ivanova, and L. A. Rescorla, "Manual for the ASEBA brief problem monitor (BPM)," *Burlington, VT: ASEBA,* pp. 1-33, 2011.

[6] L. M. v. Eijk, G. Kempen, and F. L. P. v. Sonderen, "Een korte schaal voor het meten van sociale steun bij ouderen: de SSL12-I," *Tijdschrift voor Gerontologie en Geriatrie,* vol. 25, no. 5, pp. 192-196, 1994.

[7] K. R. Bridges, R. Sanderman, and E. Van Sonderen, "An English language version of the social support list: preliminary reliability," *Psychological Reports,* vol. 90, no. 3, pp. 1055-1058, 2002.

[8] J. G. Parker and S. R. Asher, "Friendship and friendship quality in middle childhood: Links with peer group acceptance and feelings of loneliness and social dissatisfaction," *Developmental psychology,* vol. 29, no. 4, p. 611, 1993.

[9] E. Szekely *et al.*, "Childhood peer network characteristics: genetic influences and links with early mental health trajectories," *Journal of Child Psychology and Psychiatry,* vol. 57, no. 6, pp. 687-694, 2016.

[10] Netherlands Statistics, "Begrippen: Allochten," 2006.

[11] C. K. Enders, *Applied missing data analysis*. Guilford press, 2010.

[12] R. P. McDonald and M.-H. R. Ho, "Principles and practice in reporting structural equation analyses," *Psychological methods,* vol. 7, no. 1, p. 64, 2002.

[13] T. J. VanderWeele and M. J. Knol, "A tutorial on interaction," *Epidemiologic methods,* vol. 3, no. 1, pp. 33-72, 2014.
